# Clinician perspectives on explainability in AI-driven closed-loop neurotechnology

**DOI:** 10.1038/s41598-025-19510-9

**Published:** 2025-10-03

**Authors:** Laura Schopp, Georg Starke, Marcello Ienca

**Affiliations:** Laboratory of Ethics of AI and Neuroscience, Institute of History and Ethics in Medicine, School of Medicine and Health, Technical University of Munich (TUM), Ismaninger Str. 22, 81675 München, Germany

**Keywords:** Explainable artificial intelligence, Neurotechnology, Neurostimulation, Neurological disease, Psychiatric disorder, Semi-structured expert interviews, Clinical perspective, Neurology, Health policy, Medical ethics, Biomedical engineering

## Abstract

Artificial Intelligence (AI) holds promise for advancing the field of neurotechnology and accelerating its clinical translation. AI-driven clinical neurotechnologies leverage the power of non-linear algorithms to analyze complex brain data and enable adaptive, closed-loop neurostimulation. Despite these promises, the integration of AI into clinical practice remains limited, with lack of explainability being commonly cited as one main obstacle. This raises the question of whether opacity and lack of explainability also hinder the adoption of AI in closed-loop medical neurotechnologies. We investigated the attitudes, informational needs and preferences of clinicians regarding AI-driven closed-loop neurotechnologies and explored what forms of explanation they consider necessary for clinical use. We conducted semi-structured expert interviews with twenty clinicians (including neurologists, neurosurgeons, and psychiatrists) from Germany and Switzerland. Using reflexive thematic analysis, we explored their understanding of and expectations for explainability in the context of AI-driven closed-loop neurotechnology systems. Clinicians consistently emphasized the importance of context-sensitive, clinically meaningful forms of explainability such as understanding what input data were used to train the system and how the output relates to clinically relevant outcomes. By contrast, detailed knowledge of the model’s inner architecture or technical mechanics were of limited interest. Several participants specifically called for Explainable AI (XAI) techniques, particularly feature importance and relevance measures, to support their interpretation of system outputs. Our findings suggest that the clinical utility of AI-driven neurotechnologies can be improved by focusing on intuitive, user-centered and clinically meaningful forms of explainability rather than full algorithmic transparency. Designing systems that meet these pragmatic needs may help bridge the translational gap between AI development and clinical implementation.

**Supplementary Information:**

The online version contains supplementary material available at 10.1038/s41598-025-19510-9.

## Introduction

Artificial Intelligence (AI) is rapidly transforming our ability to understand and intervene in brain function and dysfunction, particularly when integrated with neurotechnology^1–4^. AI-driven tools are being increasingly explored across a spectrum of neurotechnological applications, from Brain-Computer Interfaces (BCIs) that enable motor or speech restoration in individuals with paralysis^5^ to neuromodulation therapies such as deep brain stimulation (DBS) for treatment-resistant neurological disorders^2^ or repetitive transcranial magnetic stimulation (rTMS) for psychiatric disorders^6^. Many of these technologies rely on closed-loop architectures, in which AI models continuously analyze neural signals and adapt stimulation or decoding parameters in real time. In these contexts, AI has shown promise: for example, in DBS, it can support target identification and individualized stimulation protocols by detecting complex disease patterns in large-scale neural datasets^7^. Despite these advances, the clinical integration of AI into neurotechnology is still in its infancy^7^. A key barrier in medicine is the opacity of many state-of-the-art AI models^8^. This lack of transparency poses significant challenges for clinical adoption, particularly in applications that directly interact with the human brain. In such high-stakes settings, explainability is not only a technical concern but also an ethical requirement for ensuring patient safety, preserving autonomy, and fostering trust among clinicians and patients alike.

Explainable AI (XAI) has emerged as a response to these challenges, offering tools and methods that make AI outputs more interpretable to human users^9–12^. XAI techniques such as SHapley Additive exPlanations (SHAP) can provide insights into model behavior and highlight the features most influential in AI decision-making^13^. In clinical contexts, such methods may enhance validation and support informed decision-making across medicine^14^, spanning domains as diverse as radiology, nuclear medicine, cardiology, oncology, and psychiatry^14–18^. In direct comparison with conventional, opaque AI methods, using XAI can also increase physicians’ trust in AI, as has been demonstrated in the context of melanoma diagnosis^19^. Recent research is therefore also investigating which particular XAI techniques and visualizations are perceived as useful and practical by clinicians, particularly in fields such as radiology^20^.

While XAI is gaining traction in broader healthcare settings including diagnostic software for neurology^21^, its application in medical neurotechnology—particularly in closed-loop systems—remains underexplored^22,23^. XAI implementation in the field is further complicated by the difficulty of applying standard XAI models to time series data, such as electroencephalography (EEG), as recently stressed by two reviews^24,25^. Yet, developing meaningful models of explanations for AI-driven neurotechnology is especially pressing given ethical requirements and regulatory developments such as the European Union’s AI Act, which mandates the provision of meaningful explanations for high-risk AI models involved in decisions affecting individuals^26^. These regulatory shifts underscore the urgency of understanding what constitutes a *meaningful explanation* in clinical neurotechnology contexts^27^.

This study addresses that gap. We qualitatively investigate how clinicians working with closed-loop neurotechnologies conceptualize explainability: what forms of explanation they find meaningful, useful, or necessary for clinical practice. Drawing on semi-structured interviews with twenty clinicians in neurology, neurosurgery, and psychiatry across Germany and Switzerland, we aim to map clinician-centered requirements for explainability in the context of closed-loop neurotechnologies. To our knowledge, this is the first empirical study focusing on clinical explainability needs in the context of closed-loop systems for neurological and psychiatric disorders. Our findings aim to inform technology design, regulatory frameworks, and ethical guidelines to ensure that any AI-driven neurotechnology is both clinically robust and socially aligned.

## Results

### Qualitative results

**Fig. 1:**
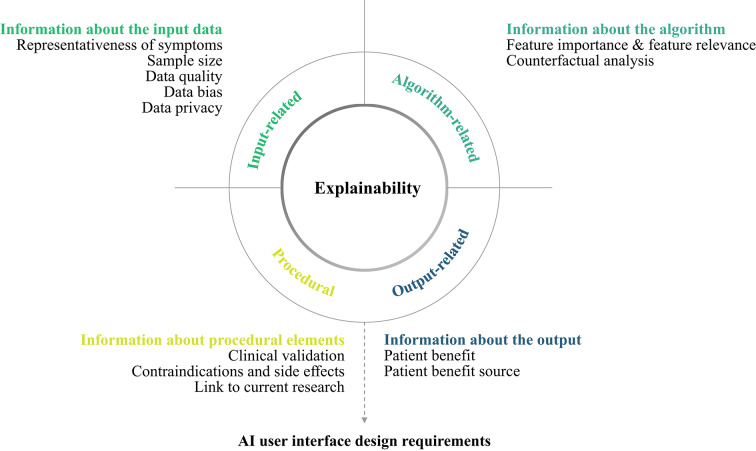
Explainability-related concept map. This explainability-related concept map illustrates the emerging themes based on our thematic analysis of the expert interviews.

Our qualitative analysis reveals a nuanced landscape of clinicians’ attitudes, informational needs and preferences concerning AI-driven closed-loop medical neurotechnology. To structure our findings, we organized clinician responses across three dimensions of the algorithmic system: its *input* (training data), its *core architecture* (algorithm), and its *output* (prediction and clinical decision). In addition, we also report their desiderates with view to user interfaces and XAI visualisations. We begin with clinicians’ views on the algorithm itself, given its centrality to current debates on explainability. The full overview of all explainability-related concepts can be seen in the concept map below (see Fig. 1).

### Algorithmic specifications and perceived relevance

A consistent theme across interviews was clinicians’ limited interest in the technical specifications of AI models embedded in closed-loop medical neurotechnological systems. Several participants (9/20) explicitly reported seeing little value in understanding technical details such as algorithm type, number of layers, or parameter counts. These aspects were generally perceived as overly technical, falling outside the their clinical expertise and responsibilities, and offering minimal practical or clinical utility for decision-making in patient care.

One participant noted that many doctors lack the expertise to distinguish between different machine learning (ML) algorithms, and several clinicians questioned whether explainability—and by extension, XAI methods—is even necessary in the context of closed-loop medical neurotechnology. Six out of twenty acknowledged the inherent opacity of AI models but expressed openness to use them nonetheless. One participant remarked that detailed understanding of the underlying AI model is rarely essential for physicians, while another observed that patients typically show little concern about algorithmic transparency. This pragmatic stance was echoed in comparisons to non-AI-driven interventions such as conventional deep brain stimulation (DBS) for Parkinson’s disease, which often prove clinically effective despite incomplete mechanistic understanding. Still, one clinician repeatedly emphasized that algorithmic transparency remains crucial for AI developers and engineers, particularly for system validation, safety assurance, and error detection. From this perspective, even if clinicians do not require direct algorithmic insight, they depend on technical and regulatory actors to ensure such understanding is firmly embedded at the system level.

### Input data information request

Our analysis reveals that clinicians’ interest in input data is shaped by underlying concerns about clinical representativeness, data accessibility, data quality, and relevance to real-world therapeutic decision-making. Several interviewees highlighted the critical role of knowledge about input data in shaping clinicians’ trust in, and expectations of, future AI-driven medical closed-loop neurotechnologies.

#### Representativeness of training data

Clinicians (4/20) voiced scepticism about whether the full spectrum of neurological and psychiatric symptoms can be adequately captured by training datasets. In disorders such as Parkinson’s disease, where symptoms such as tremor vary widely across individuals, participants stressed the need to understand whether the AI models had been trained on data representative of their own patient populations.

#### Access to input data

Some research-oriented clinicians (3/20) emphasized the need for transparent access to the raw data that underpin AI decisions, to better understand AI and determine its applicability. Especially in high-stakes contexts like critical care, they expressed a desire to inspect and interpret input signals independently. This demand for data transparency was closely linked to clinicians’ need to validate AI-driven interventions and retain clinical oversight, especially when the treatment mechanism is not fully explainable even without AI.

#### Multimodal input and new biomarker

Multiple interviewees agreed (4/20) that neural activity data alone is insufficient for building and implementing high-performing closed-loop systems. Clinicians strongly advocated for the integration of complementary inputs such as wearable sensor data, video-based movement assessments, and subjective patient-reported outcomes, including measures of quality of life. One clinician even remarked that therapeutic efficacy does not require full mechanistic understanding, so long as the patient’s well-being improves. Consequently, clinicians also expressed a desire to know whether multimodal and subjective patient data was used to train a specific AI system. At the same time, four clinicians noted that certain biomarkers for neurological and psychiatric conditions are already too complex to be fully understood, even without the use of AI, with two of them reporting that parameter settings in some neurostimulation devices are often determined through trial and error. This highlights a shift toward outcome-oriented validation and supports the integration of multimodal data pipelines in AI system design.

#### High data quality and generalizability

Three out of twenty clinicians raised concerns about the noise and artifact susceptibility of brain data, particularly when acquired under clinical conditions. They emphasized the need for robust preprocessing pipelines and artifact removal to ensure that algorithms learn from signal rather than noise, and demanded that appropriate steps are reported. Additionally, one participant questioned the adequacy of existing datasets, citing small sample sizes and variability in patient condition, device configuration, and electrode placement as potential limitations.

### Key information about the output

Clinicians expressed their strongest concerns (11/20) about the output of AI-driven systems, particularly in terms of safety, patient benefit and clinical relevance, and connected this to their informational needs and preferences. Many emphasized that their trust in AI is shaped not by insight into the algorithms themselves, but by the real-world consequences of their decisions and actions.

#### Safety of the output and operational transparency

Another concern raised by one clinician was the safety of algorithmic outputs, especially in scenarios where systems can autonomously adjust neurostimulation parameters in real time. Four out of twenty clinicians emphasized the need to understand not only the system’s accuracy, but also how its output is operationalized and translated into clinical action. This concern was particularly acute in sensitive or unpredictable environments involving potential real-time interventions, such as seizure detection by responsive neurostimulation (RNS) systems. Seven out of twenty clinicians noted the importance of clearly defined safety boundaries to prevent outputs that could result in harmful or socially dangerous actions (e.g., during driving or unsupervised movement). Several participants stressed that AI models adjusting stimulation parameters autonomously must be constrained by hard safety limits, ensuring that output decisions cannot exceed clinically safe thresholds.

#### Alignment with clinical reasoning and evidence of patient benefit

Clinicians were more likely to trust AI models when the system’s recommendations were aligned with their clinical reasoning or “*gut feeling*”. In their view, trust was not gained through detailed algorithmic explanations, but rather through consistent congruence between AI recommendations and clinician intuition. Although most clinicians expressed optimism about AI’s future role in closed-loop systems, they voiced frustration at the current gap between technical potential and clinical application. Some (3/20) described the field as “overhyped,” arguing that claims of transformative impact lack sufficient empirical support. For example, one clinician cautioned that adding adaptive features to DBS treatment is unlikely to improve by more than 30%. While acknowledging the strengths of AI in data processing and predictive modelling, participants consistently emphasized that tangible patient benefits must be demonstrated through realistic, clinically grounded trials. Several noted that new implantable pulse generators (IPGs), which enable long-term neural data collection, present a valuable opportunity to generate the kind of real-world datasets needed to rigorously assess AI’s clinical utility. Ultimately, participants underscored that AI should be deployed only when it leads to clear, demonstrable improvements in patient outcomes, and that these benefits must be carefully evaluated through transparent and ethically sound clinical trials.

#### Meaningful clinician-AI interaction and clinical relevance

Eight participants emphasized that AI should support, not replace, clinical judgment. One clinician cautioned that AI models might identify statistically significant patterns that lack medical validity, reinforcing the need for clinicians’ oversight in defining use cases and validating model outputs. Several clinicians also emphasized that AI systems require clear hypotheses to address real clinical problems and cannot generate meaningful solutions in isolation.

### AI user interface design requirements

Clinicians provided valuable insights on the design of user interfaces for AI-driven closed-loop neurotechnology when prompted, emphasizing the importance of intuitive, context-specific visualizations that support understanding without demanding technical expertise in ML.

#### Data transparency through visualization

There was broad agreement among participants (9/20) that descriptive statistics and visual summaries, such as charts, enhance understanding of the training data. These tools were considered essential for assessing the representativeness and relevance of the dataset to individual patient cases. One clinician suggested incorporating visualizations of symptom clusters to verify whether an individual patient matches specific subgroups in the training set.

#### Linking to scientific evidence

A clinician welcomed the idea of embedding hyperlinks to peer-reviewed publications that underpin model decisions. Such links could strengthen trust by showing that algorithmic outputs are grounded in established clinical knowledge and by allowing users to verify the underlying medical rationale.

#### Explainability tools

Only three out of twenty participants spontaneously expressed interest in formal XAI methods such as feature relevance, feature importance rankings, and counterfactual examples. Those who did valued the ability to identify top predictors or explore counterfactuals and *what-if* scenarios. One participant also called for access to source data within the interface, particularly for research or validation purposes. Interestingly, two clinicians preferred paper-based formats over digital dashboards when reviewing complex patient profiles, suggesting that interface flexibility remains crucial.

#### The limits of visualization

Despite general appreciation for transparency tools, several participants cautioned that data visualization alone does not fully resolve the challenges of algorithmic opacity. As a result, clinicians advocated not for complete transparency but for *practical intelligibility*: interfaces that convey enough information to safely and confidently integrate AI support into clinical workflows, without overwhelming users with unnecessary complexity.

Illustrative theme-specific quotes are presented in Table 1.

**Table 1:** Illustrative quotes for the respective themes.

Algorithmic specifications information request	
	“To be honest, as a doctor I don’t feel comfortable to really understand the differences between a random forest and a Support Vector Machine (SVM).” (P6)
	“I don’t know whether I need to understand what the AI model is doing.” (P11)
	“Well for the patients [AI and training data] is not relevant, I mean for 99,9% of all patients. But for me it is interesting […].” (P12)
	“As with any technical solutions, there needs to be someone who can repair it […]. This is the same for AI-driven technology, so I believe that engineers and technicians need a good understanding of how to repair it and decide when to decommission it.” (P18)
	“But verifying how the machine or the technology has derived the result, I think, is [for me] overrated. […] I think that’s certainly a different question for engineers and developers.” (P18)
Input data information request	
	“[…] There are a lot of clinical specifications, so therefore I would like to know if the training data is suitable to be used for certain situations.” (P12)
	“[…] But there are a lot of [tremor] subtypes, e.g., for Parkinson’s disease patients this means many axial symptoms, and these are more difficult to be reduced by neurostimulation.” (P10)
	“We need unrestricted access to the source data. We simply need to be able to obtain and analyze this source data without any time delay and, if necessary, to be able to model it with AI to really make a statement, as long as we don’t have this, we are a bit in a black box situation and only see the AI-driven output at some point […]. But that’s the important thing.” (P19)
	“Closed-loop plays a big role in the sensory and motor symptoms domain. And in this area, we would say the main thing is it works, and the patient has a better quality of life.” (P7)
	“Because we really don’t know, so it’s sometimes more like Frankenstein research, you try it and see what happens.” (P7)
	“Reality is much more complex than all the, I'll say, training datasets you have and what looks great and works great in training datasets, as soon as you let a little more reality it starts to collapse.” (P7)
	“We did a project with a start-up from the [blinded]. We wrote a Science paper together where, all of a sudden, a hormone level was statistically significant for the algorithm’s prediction. This was complete nonsense from a clinical perspective (although the data scientist liked the result) because this hormone level was a proxy for a certain medication for only very sick patients”. (P13)
Output information request	
	“Even if you don’t understand the system itself, you might at least be able to assess the consequences of using it.” (P7)
	“With DBS, we have an extremely efficient therapy and all that without AI. The thing is, DBS is not going to improve by 30% because we all of the sudden have adaptive DBS […].” (P15)
AI user interface design requirements	
	“If the focus is too broad, this might lead to a cancelling out of the effects.” (P10)
	“Just link the relevant publications to the findings to see the robustness of the features in other research as well.” (P13)
	“As a researcher, I would like to have 100% transparency and access to the data.” (P7)

## Discussion

These findings provide novel insights into how clinicians engage with, evaluate, and ultimately decide whether to trust AI-driven closed-loop medical neurotechnologies. This exploration of expectations around input data, algorithmic transparency, algorithm output, and interface design, offers a comprehensive view of what explainability means to clinicians. Two critical insights emerge from our study. First, clinicians’ trust in AI and perceived clinical utility of AI-driven neurotechnology do not depend on full algorithmic transparency but on the provision of context-sensitive, clinically meaningful forms of explainability. This shifts the emphasis from traditional XAI goals toward more pragmatic, user-centered approaches tailored to clinical reasoning.

Second, our interviews revealed that explainability is not viewed by clinicians as a static technical property but rather as a dynamic and context-sensitive component of decision-making. Trust in AI models, therefore, is not built through detailed disclosures of algorithmic architecture but through system behaviours that align with clinical logic and patient outcomes. An emerging concern in this context was the tendency of AI models to learn and rely on epiphenomena, i.e. statistical artefacts or spurious proxies, rather than underlying pathophysiological mechanisms. This can result in seemingly accurate predictions that, while technically valid within the dataset, are clinically irrelevant or misleading. For instance, one clinician recounted a case where an AI model identified a hormone level as statistically significant for predicting an outcome, despite it being merely a proxy for medication use in severely ill patients. From a medical standpoint, this result was deemed to be “complete nonsense”, yet the data scientist regarded it as an interesting signal. Such cases exemplify the epistemic fragility of AI outputs when interpretability is lacking and when models exploit correlations that do not correspond to causal or physiologically meaningful relationships. This challenge underscores the importance of grounding explainability not just in clarity or transparency, but in the clinical meaningfulness and plausibility of the model’s outputs. In this high-stakes context, it is critical to distinguish between correlation and causation and to ensure that models reflect mechanisms clinicians can reason about and act upon. In broader terms, our findings reveal that clinicians want to know what data the system uses and how the output is relevant to their clinical goals, rather than how the algorithm operates internally. This challenges the prevailing one-size-fits-all model of XAI and points instead to the importance of designing neurotechnologies that embed intelligibility over interpretability.

Although most participants showed little interest in technical specifications or traditional XAI tools, this should not be mistaken for disinterest in transparency altogether. On the contrary, clinicians valued outputs that could support clinical reasoning such as feature importance scores, confidence estimates, or ranked outputs that align with medical judgment. In many cases, clinicians compared AI models to other medical tools whose mechanisms are not fully understood but are trusted based on demonstrated utility and effectiveness. This view resonates with London et al.’s observation that “[a]s counterintuitive and unappealing as it may be, the opacity, independence from an explicit domain model, and lack of causal insight associated with some of the most powerful ML approaches are not radically different from routine aspects of medical decision-making.”^28^. This challenge is further compounded by a fundamental distinction between neurotechnology and other clinical applications of AI. In more established clinical domains, such as outcome prediction or diagnostic support, the model inputs are themselves inherently interpretable, hence clinicians are already familiar with variables such as patient age, blood pressure, lab values, or imaging findings. In these cases, the primary opacity lies in the model’s internal reasoning, which XAI tools aim to elucidate. In contrast, neurotechnology introduces an additional layer of opacity: the input signals themselves, such as raw neural time series or local field potentials, may lack immediate clinical interpretability. Clinicians are not typically trained to interpret electrophysiological waveforms or spectral decompositions, nor are these signals intuitively linked to clinical symptoms or outcomes in the same way that, for instance, hyperglycemia might be linked to diabetic complications. This *dual-layer opacity* at both the input and algorithmic levels poses unique challenges for explainability and limits the applicability of many standard XAI techniques developed for structured or tabular data. A good example of that is closed-loop DBS, where clinicians routinely operate without full mechanistic understanding at neither input nor algorithm level. This underscores the need for novel, domain-specific explanation strategies that not only clarify the model’s decision pathway but also translate raw neurophysiological data into clinically meaningful concepts. From a design perspective, this means AI models must support *functional intelligibility*, not just *interpretability*. For example, while feature importance and SHAP values can be helpful, they may not be sufficient in isolation. Several participants proposed the development of interactive explanation tools, including LLM-based chatbots, that could engage clinicians in two-way conversation and adapt explanations to their level of expertise or context of use. Such tools could help bridge the gap between the technical language of AI developers and the clinical language of medical professionals. This gap that was repeatedly identified as a key barrier to trust.

Clinicians also emphasized that trust in AI systems for neurotechnology is ultimately earned through demonstrated performance, safety and relevance to patient care. They concurred that systems that consistently offer accurate, safe, and clinically relevant recommendations are more likely to gain acceptance, even if their inner workings remain partially opaque. Importantly, they cautioned that AI-generated outputs such as DBS stimulation parameters must be subject to the same scrutiny and regulatory oversight as any other clinical intervention, including drugs and other medical devices. Furthermore, algorithmic decisions must not bypass clinical accountability, regardless of their origin (see Table 1 for illustrative theme-specific quotes).

It is possible that the participants’ preference for user-centric framings of explainability also reflects the current stage of the field. Clinicians noted that AI remains underutilized in closed-loop medical neurotechnologies, and that the absence of validated, real-world systems limits both practical experience and demand for explainability. In this sense, the underuse of AI and the limited emphasis on explainability may reflect the same root cause: a lack of functional, validated AI applications in routine clinical practice. Many interviewees observed that the field is still in its early stages: many IPGs are just beginning to generate the large-scale data needed to train and validate ML models. Without such data, clinically meaningful AI applications remain underdeveloped, and questions of explainability remain largely theoretical. Open data concepts were also welcomed by the participants. However, the expectation for open access to training data was not considered always realistic in the current commercial and legal landscape. Companies may be reluctant to disclose proprietary data due to intellectual property concerns, competitive advantage, or compliance with national data protection laws, including regulations that restrict cross-border data sharing. These constraints highlight the need for balanced governance frameworks that can uphold the legitimate interests of stakeholders while still promoting sufficient transparency and trustworthiness for clinical deployment.

Participants also acknowledged clinicians’ limited technical training in AI and how this can challenge their efforts to seek explanations that are both clinically meaningful and actionable. This points to a dual responsibility: while AI developers are increasingly expected to design interfaces and systems that balance explainability with clinical relevance, clinicians—and the institutions that train them—may bear a corresponding responsibility to adapt medical education in ways that strengthen physicians’ capacity to interpret and critically engage with AI systems*.*

Several additional barriers to trust in AI-powered neurotechnology were also identified, including restricted access to input data. Clinicians expressed frustration that manufacturers often withhold valuable training datasets, which stifles collaborative validation and hinders transparency. This asymmetry in data access not only inhibits collaborative validation efforts but also impedes trust and limits feedback loops essential for continuous improvement. Bridging this gap will require incentives for data sharing, regulatory clarity, and the development of robust, testable proof-of-concept systems clinicians can evaluate firsthand.

Our findings have direct implications for AI design in neurotechnology settings. Bridging the gap between clinicians’ informational needs and the technical feasibility of current XAI methods clearly emerged as an open challenge for medical AI development. In light of our findings, one promising approach may be the use of hybrid explainability methods that combine statistical techniques (e.g., SHAP values, saliency maps) with case-based or example-based reasoning, such as presenting similar patient trajectories or prototypical cases to contextualize algorithmic outputs. These strategies align more closely with clinicians’ existing cognitive models and decision-making processes. Equally important is the translation of empirical insights (including those collected in this study) into concrete design features, such as adaptive user interfaces that surface different levels of explanation depending on user expertise, or workflow-integrated visualizations that prioritize clinical relevance over algorithmic transparency. Embedding such human-centred considerations at the interface or system design stage may help ensure that XAI tools serve clinicians’ real-world needs rather than abstract technical ideals and may help reconcile technical opacity with the demand for clinical trustworthiness and oversight. Further research is needed to assess how these design principles can be operationalized across diverse clinical settings and neurotechnological applications.

Our findings also carry implications for ongoing AI policy development. By highlighting clinicians’ preference for context-sensitive, functionally intelligible outputs over full algorithmic transparency, our results suggests that regulatory frameworks such as the EU AI Act must account for domain-specific expectations of explainability that reflect real-world clinical reasoning, where trust is shaped more by outcome alignment and system reliability than by detailed technical interpretability. At the same time, clinical usability should not be the sole determinant of regulatory explainability standards. The AI Act rightly emphasizes *transparency* and the provision of “*meaningful information”* to users, while also enshrining key patient rights such as the right to explanation, human oversight, safety, and protection from harm. Especially in high-risk applications like AI-enabled neurotechnologies, these rights underscore the importance of explanations that extend beyond clinical workflows. While clinicians may be satisfied with pragmatic, outcome-focused explanations that support decision-making, patients may require different forms of understanding, particularly when interventions involve autonomous systems that affect their mental or physical integrity. Accordingly, physician-centered explainability can enhance adoption and clinical implementation but may not fully circumscribe the explanatory arena for AI-driven medical management.

It should be emphasized that clinicians’ pragmatic preference for functional intelligibility does not seem to negate the ethical imperative associated with AI-driven neurotechnologies. On the contrary, the tenet that clinical neurotechnology, especially in implantable and closed-loop forms, constitutes a high-stakes setting is widely acknowledged among our participants and in the bioethics and neuroethics literature. Rather, this divergence underscores a crucial governance challenge: while clinicians may be satisfied with lean, context-specific forms of explanation, such forms may not be sufficient to address the full spectrum of ethical concerns posed by neurotechnological interventions. This raises another ethical and policy question: is functional intelligibility (however adequate it may be for clinical decision-making) sufficient for ensuring ethical accountability in the development and deployment of AI-enabled neurotechnologies? Especially in light of existing regulatory frameworks like the EU AI Act, the limits of physician-centered explainability must be critically examined.

One possible solution is prioritizing tiered or role-adapted explainability frameworks^29^, which tailor different types of explanations to different stakeholders; for instance: functional clarity for clinicians, accountability mechanisms for regulators, and rights-based justifications for patients. Policymakers could sustain these efforts by prioritizing flexible, stakeholder-informed approaches to explainability that reconcile ethical imperatives with practical constraints. Such approaches can enhance trust while ensuring that AI regulation meaningfully safeguards patient autonomy and well-being, without unduly impeding innovation in and clinical translation of valuable AI-based medical technologies (see Table 2).

**Table 2:** Recommendations for technology designers and developers of AI-driven closed-loop neurotechnologies.

Design Focus	Recommendations	Rationale
Explainability	Prioritize clinically relevant explanations (e.g., input–output logic, feature importance)	Clinicians value understanding how inputs relate to outputs over technical model details
User-centered interfaces	Design interfaces that visualize AI outputs and relevant features in an intuitive clinical format Prioritize tiered and role-adapted explainability frameworks	Supports rapid interpretation and integration into clinical workflow Promotes the provision of information based on stakeholder-specific informational needs and ethical priorities
Transparency over full disclosure	Offer selective transparency tailored to user needs rather than full algorithmic transparency	Full technical detail is often irrelevant; actionable clarity is more effective
Context-specific XAI tools	Implement explainability methods such as SHAP adapted to the neuroclinical use case	Clinicians responded positively to familiar, task-specific interpretability tools
Clinical relevance assurance	Ensure outputs align with clinical goals, terminology, and decision pathways	Builds trust and promotes usability by linking AI reasoning to real-world clinical logic
Iterative co-design	Involve clinicians throughout the development lifecycle	Incorporates real-world constraints and enhances acceptance through early stakeholder input
Ethical and regulatory alignment	Embed explainability features that meet legal standards and protect patient rights (e.g., EU AI Act, Article 86)	Ensures compliance and mitigates future policy and liability risks

## Methods

We conducted semi-structured interviews with German-speaking clinicians working with closed-loop neurotechnologies for neurological and psychiatric conditions. The sample comprised 20 clinicians: 13 neurologists, 5 neurosurgeons, and 2 psychiatrists based in Germany (n = 16) and Switzerland (n = 4). Participants’ clinical and academic ranks ranged, respectively, from resident physician to head of department, and from Doctor of Medicine (MD or Dr. med.) to Full Professor. After 20 interviews we achieved data saturation, the point at which no new information or themes emerge from the dataset, hence further data collection is unlikely to add value to the analysis and is therefore deemed unnecessary^30^. Consequently, we stopped recruitment of additional interview candidates thereafter. We identified participants using purposive snowball sampling. Initial candidates were selected through systematic screening of high-impact publications, institutional websites, and the German DBS registry (“THS-Zentren”) maintained by the *Arbeitsgemeinschaft Tiefe Hirnstimulation.* Additional interviewees were recruited via expert referrals. Inclusion criteria included a medical degree, at least one publication in the field, and clinical experience in one of the relevant specialties. Efforts were made to ensure diversity in gender, career stage, and institutional affiliation.

Interviews were conducted between August 2024 and January 2025 by the first author, a PhD candidate in biomedical ethics with a degree in data science. The interview guide was collectively developed by our team based on previously validated research protocols^31^. Prior to data collection, a pilot interview with a board-certified psychiatrist and neuroethics researcher, served as an internal pilot. Following six interviews, the interview guide was reviewed by the second and third author. This led to minor adaptations such as adding a prompt concerning the trade-off between algorithmic information and hands-on clinical familiarity with neurostimulation devices. All interviews (n = 20) were conducted in German via online call (n = 13), phone (n = 6), or in-person (n = 1). Interview duration varied between 20 and 43 min (mean duration: 34 min). Recordings were transcribed locally using MacWhisper software (version 2.20 (121)) and manually corrected for accuracy by the first author. Relevant excerpts were translated into English and independently reviewed by the second author. Thematic analysis was performed using a reflexive approach, and themes were inductively developed, labeled in English, and iteratively reviewed by the second and last author to ensure coherence and validity.

Prior to data collection, we obtained, on 1 March 2024, an ethics waiver (Unbendenklichkeitserklärung) for the expert interviews from the competent medical ethics committee at the Technical University of Munich. To adhere to established ethical standards, participants received an information sheet, provided written informed consent, and gave verbal consent to audio recording. Transcripts were de-identifyied and securely stored on an encrypted local device. The study was conducted in accordance with the Declaration of Helsinki and all relevant institutional and national research regulations. An overview of the participants gender, origin, main discipline and career level can be found in Table 3.

**Table 3:** Participant characteristics.

Characteristics		Participants N = 20
Gender n (%)	Male	18 (90)
	Female	2 (10)
Country n (%)	Germany	16 (80)
	Switzerland	4 (20)
Main discipline n (%)	Neurology	13 (65)
	Neurosurgery	5 (25)
	Psychiatry	2 (10)
Career level n (%)	Professor	9 (45)
	Head of Department	2 (10)
	Attending Physician/Consultant	8 (40)
	Resident physician	1 (5)

While this study provides valuable insights into clinicians’ perspectives on explainability in AI-driven closed-loop medical neurotechnology, several limitations must be acknowledged. As with all qualitative research, the findings are not statistically generalizable and reflect the beliefs, attitudes, and experiences of a specific group of twenty German-speaking clinicians from Germany and Switzerland. Despite efforts to achieve gender balance, female participation was limited. Of the 26 female clinicians contacted, only two agreed to participate in the study (rounded 8% participation rate), compared to a 17% participation rate among male clinicians. Additionally, this study provides insights into end-user requirements in the context of requirements engineering, but is not a proof-of-concept (PoC) of AI or XAI methods in the closed-loop medical neurotechnology. Furthermore, the primary interviewer’s expertise in the field may have subtly shaped the interview dynamics, potentially influencing participants’ responses through implicit cues or shared assumptions. While this positionality can enrich thematic depth, it may also introduce bias. Finally, although all expert interviewees work directly with closed-loop medical neurotechnology, their technical understanding of AI and specific XAI likely varies. However, the aim of this study was not to evaluate technical proficiency in AI or XAI, but to explore clinician-centered end-user requirements without the need for a deep technical understanding of AI or XAI. We believe that this perspective remains critical for informing responsible and usable system design, and that these limitations do not diminish the novelty or relevance of our findings.

## Conclusion

Our findings highlight the need for a paradigmatic shift in how explainability is approached in AI-driven closed-loop neurotechnology. Rather than aiming for full algorithmic transparency through exhaustive XAI methods, developers should prioritize intuitive, clinically meaningful forms of explanation that align with how clinicians think, decide, and act. This demands moving beyond static technical definitions of explainability and embracing a more contextual, pragmatic design philosophy that considers the informational needs of end users such as the representativeness of symptoms in the input data and the clinical relevance of the output. Explainability should not be an afterthought or a one-size-fits-all checklist item. It must be embedded across system design: from transparent and collaborative data practices to interpretable and safety-conscious outputs, to interfaces that facilitate clinical reasoning through adaptive, user-specific interactions. Policymakers, too, must recognize that meaningful transparency is inherently relational, i.e. shaped by the user, the task, and the clinical setting. Regulatory frameworks should therefore support not just generic disclosure, but the development of clinically validated AI models that earn trust through demonstrable performance, clear benefits, and accountability mechanisms that mirror established clinical standards. Additionally, translating the promise of AI into real-world clinical impact will depend on designing systems and more precisely end-user interfaces that clinicians do not merely understand but can confidently rely on throughout medical decision-making. To ensure the clinical viability of XAI methods in AI-driven closed-loop neurotechnology, our conceptual findings need to be translated to testable metrics, performance benchmarks, and technical features as well as to technical infrastructure that enables the kind of transparency that clinicians desire. Ultimately, future research efforts should also include patients’ perspectives and expectations regarding AI and its explainability to preserve patient autonomy and foster trust.

## Supplementary Information

Below is the link to the electronic supplementary material.

Supplementary Material 1

Supplementary Material 2

## Data Availability

The coded and deidentified quotes on which our findings are based have been made publicly available at Zenodo: https://doi.org/10.5281/zenodo.16528872.
