# Supplementary material for: Clinician perspectives on explainability in AI-driven closed-loop neurotechnology
Source: Sci Rep. 2025 Oct 3;15:34638. doi: 10.1038/s41598-025-19510-9 (PMC12494947; doi:10.1038/s41598-025-19510-9)
Supplement: Supplementary file 1 — Supplementary Material 1 [file 41598_2025_19510_MOESM1_ESM.docx]

Additional file 1: Interview guide

| Part of the interview guide / theme / technique | Interview question | AIM: What should be dealt with |
| --- | --- | --- |
| **Introduction** | - Can you tell me a little bit about your work?   - What is your area of expertise/specialization?   - Please describe your roles and responsibilities, including any research or clinical commitments?   - What projects are you currently involved in? | - Ice breaker - Getting to know daily work of the clinician |
| **Neurotechnology** | - What **role does neurotechnology play in your current work**?   - Which specific systems do you work with?/Which specific system are you most familiar with?   - What kind of patients do you typically work with using these systems? [Maybe asking about the characteristics of the patients]   - What is your specific role in the application of the system for treatment?   - Does the system you work with operate on a closed-loop basis? How would you define [UPDATE BASED ON SYSTEM] a closed-loop system? | - How relevant is neurotechnology for the clinician? - What is his/her level of expertise? |
| **Neurotechnology and AI** | - What are your thoughts on the integration of AI models in neurotechnology?   - What advantages and potential concerns do you see with the use of AI models in this field?   - Are you aware of any AI model used/applied in the neurotechnology you are using/implanting?/Are you aware of any AI model used/applied in neurotechnology devices? - Do you have any specific AI use cases for [UPDATE BASED ON SYSTEM] in mind?   - What would be the benefit of using AI in this use case?   - What specific tasks do you envision AI models performing for you?   - Imagine you are working with the system [UPDATE BASED ON SYSTEM], what would be the one thing you want an AI model to do for you? | - Getting an understanding of their beliefs towards AI. - What are the challenges the clinicians are currently facing? |
| **Neurotechnology and XAI** | - In your **role as physician/decision-maker, do you think information about the AI models prediction/classification needs to be provided**?   - If so, what kind of information would you consider adequate/appropriate?     - Information on the training datasets?     - Information concerning the AI model itself?     - Information on the accuracy of the AI model?     - Information on the safety of the AI model? - Do you think that an AI model should provide some explanation or justification for their recommendations?   - If so, what kind of explanation/justification would you consider sufficient for using this medical AI system in your clinical practice? - **What are the ethical implications** of faulty/invalid AI model explanations? - Would you prefer an AI model that offers detailed explanations for its recommendations, or one that is merely certified by regulatory bodies? - Do you consider any information about the AI as relevant or do you not care so much about any information because you want to clinically test the AI-based device yourself? - Would you hesitate to use/prescribe your system if it contains AI-based components that you cannot understand?   - Would you change your mind if the AI model and ultimately the system was certified by the regulatory authorities without any further explanation on why the system got its approval? - In what way would you want to receive information about the AI models justification (e.g., digital/non-digital documentation, chatbots, hotlines, training etc.)? - Would you prefer the justifications to include visuals (diagrams, charts, illustrations) or text or both? | - Getting an idea of the **clinicians understanding and relevance of explainability.** - What are the end-user requirements of explainability? - What is the most important information they need to trust the system? |
| **Closing up** | - **How do you see your role in the future working together with AI, are you optimistic/concerned?** - Is there any other topic that you would like to discuss which we have not yet covered? - Is there anything you would like to add? | - **Perceived chances and risks of AI in their field.** |
